# Real-time determination of flowering period for field wheat based on improved YOLOv5s model

**DOI:** 10.3389/fpls.2022.1025663

**Published:** 2023-01-11

**Authors:** Xubin Song, Lipeng Liu, Chunying Wang, Wanteng Zhang, Yang Li, Junke Zhu, Ping Liu, Xiang Li

**Affiliations:** ^1^ College of Mechanical and Electronic Engineering, Shandong Agricultural University, Taian, China; ^2^ School of Agricultural and Food Engineering, Shandong University of Technology, Zibo, China; ^3^ State Key Laboratory of Crop Biology, College of Life Sciences, Shandong Agricultural University, Taian, China

**Keywords:** field, wheat, machine vision, YOLOv5, flowering period determination

## Abstract

The flowering period is one of the important indexes of wheat breeding. The early or late flowering affects the final yield and character stability of wheat. In order to solve the problem that it is difficult to accurately and quickly detect the flowering period of a large number of wheat breeding materials, a determination method of flowering period for field wheat based on the improved You Only Look Once (YOLO) v5s model was proposed. Firstly, a feature fusion (FF) method combing RGB images and corresponding comprehensive color features was proposed to highlight more texture features and reduce the distortion caused by light on the extracted feature images. Second, the YOLOv5s model was selected as a base version of the improved model and the convolutional block attention model (CBAM) was adopted into the feature fusion layer of YOLOV5s model. Florets and spikelets were given greater weight along the channel and spatial dimensions to further refine their effective feature information. At the same time, an integrated Transformer small-target detection head (TSDH) was added to solve the high miss rate of small targets in wheat population images. The accurate and rapid detection of florets and spikelets was realized, and the flowering period was determined according to the proportion of florets and spikelets. The experimental results showed that the average computing time of the proposed method was 11.5ms, and the average recognition accuracy of florets and spikelets was 88.9% and 96.8%, respectively. The average difference between the estimated flowering rate and the actual flowering rate was within 5%, and the determination accuracy of the flowering period reached 100%, which met the basic requirements of the flowering period determination of wheat population in the field.

## 1 Introduction

Flowering period reflects the growth of various crops. The flowering period of wheat determines the growing period of wheat, which marks the transition from nutrient consumption to nutrient accumulation, and then influences the final yield and the stability of traits (Wang et al., 2020; Velumani et al., 2020; Zhu et al., 2021). Different varieties of wheat have different flowering periods and flowering traits, and the flowering situation is also changed of the same wheat varieties grown at different locations (Liu et al., 2015). The genotypic performances of different varieties under such conditions as diverse environments and planting practices can be determined by recording the flowering period of wheat. At present, the flowering period of a large number of wheat breeding materials and germplasms is mainly determined by manual estimation of the proportion of florets to spikelets, which is labor-intensive, time-consuming, and subjective (Zhang et al., 2020). Therefore, it is necessary to study an efficient and accurate detection technology to replace manual labor and provide critical information for the development of wheat germplasms.

The technology of plant phenotype monitoring has been called the fourth revolution in agricultural production (Sishodia et al., 2020). Efficient, automated, and versatile phenotypic techniques are important tools for accelerating the breeding process and improving genetic gain (Zhao et al., 2019). Phenotypic acquisition built on machine vision is a fast, low-cost, non-destructive technique that can achieve accurate extraction of target traits (Hu et al., 2019; Ajlouni et al., 2020; Zhang et al., 2020). With the development of imaging equipment and image processing algorithms, machine vision technology has made tremendous advances in detecting plant flowering periods. At present, many research results on flowering detection of fruit trees, shrubs, and other plants. Xiong et al. (2021) realized the instance segmentation of lychee flowers based on the DeepLab V3 network model. The recognition accuracy of the network model reached 87.0%, and the detection time of a single image was 67ms. Zhao et al. (2020) proposed a tomato flowering detection method based on the cascading convolutional neural network to identify tomato flowers at different flowering stages. The average recognition accuracy of the proposed method was 82.8%, and the detection time of a single image was 12.5ms. Most of the above studies were conducted on fruit trees and shrub plants with low planting density and large differences in flower characters, leaves and background. In a complex field environment, Milicevic et al. (2020) solve the problem of mutual occlusion among corn plants by installing hundreds of automatic cameras in the experimental field to collect images parallel to the top of each corn. In order to realize the detection of corn flowering, the main spike of the tassel was segmented based on the image processing method, and the recognition accuracy of complex targets was improved by using the cross-entropy loss function of dynamic scaling. The average recognition accuracy of the maize tassel was improved to 91.1%. Cai et al. (2021) proposed a method to determine the flowering period of the sorghum based on multi-temporal spike counting to realize flowering period detection in the field environment. The YOLOv5 model was used to detect and count sorghum spikes, and the average recognition accuracy was 86.2%. The proposed method was able to accurately detect sorghum spikes and calculate the flowering period. However, the above studies did not rule out the possibility that the influence of light conditions on the determination. When the reflection of the wheat spike was obvious, the recognition accuracy would be affected.

Over the years, scholars have explored some computer vision detection methods to study the flowering period of wheat. Sadeghi-Tehran et al. (2017) presented an automated method to detect wheat heading and flowering stages. The bag-of-visual-word technology was used to identify the flowering of wheat ears in digital images and determine whether the wheat in the image is in the flowering stage. The accuracy rate of flowering detection was 85.45%. Ma et al. (2020) proposed a two-stage segmentation method based on superpixel clustering and the fully convolutional network (FCN) to realize the segmentation of wheat spikes of the wheat canopy image at the flowering period. The accuracy of flowering spikes segmentation was 83.7%. The above studies were to realize the recognition of flowering wheat by importing color and texture features into the Support Vector Machine (SVM) for training, or using the convolutional neural network to train labeled flowering ears and non-flowering ears. However, the existing research only identified the flowering and non-flowering wheat ears and did not determine the flowering period of wheat in the field, which could not provide objective and comprehensive data.

The flowering period of wheat in the field is mainly determined by the proportion of florets to spikelets. Compared to other crops, the florets and spikelets of wheat have small morphological structures and the color differences are not obvious (Bommert et al., 2005; Cheng et al., 2016), which increased the difficulty of detection. In particular, there are the following difficulties to realize flowering time detection of wheat populations in the field environment: Firstly, the environmental background is complicated, and common image pretreatment methods cannot globally suppress noise from light, wheat awns, leaves and soil; secondly, when wheat enters the flowering period, the leaf extension is large, and the spikelets will overlap with each other. Finally, florets and spikelets belong to small-scale targets in the population of images, and the scale changes drastically according to the different shooting distances.

Therefore, to achieve an accurate determination of the flowering period in wheat, firstly, we proposed an image enhancement method based on feature fusion, which reduces the light distortion of wheat images and highlights more texture features. On this basis, an improved YOLOv5s model was proposed to optimize the extraction efficacy of obscured wheat spikes, and solve the problem of the high detection rate of small target leakage in wheat population images. Finally, the accurate and fast detection of florets and spikelets was achieved, and the ratio of florets to spikelets was used to determine the flowering period. It can provide data support for wheat stable yield improvement.

## 2 Materials and methods

### 2.1 Data acquisition

**Figure 1:**
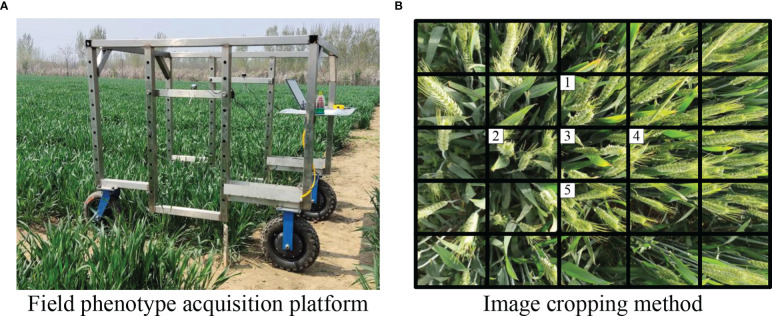
Wheat image data acquisition. **(A)** Field phenotype acquisition platform. **(B)** Image cropping method.

The images were taken at the Agricultural Experimental Station in Shandong Agricultural University, Taian, China [36°9′52″N, 117°9′21″E]. During the shooting period, the weather was mostly sunny, with less cloudy and rainy days. The image data were acquired by the data acquisition platform as shown in **Figure 1A**. The Jierui Weitong DW800_2.9mm camera(Lens: no distortion, wide angle 2.8mm; Angle: Oblique 45 degrees; Shutter speed: 1s) was mounted on the side of the Phenotype platform, 1m above the ground. The camera manufacturer is Shenzhen Jerui Weitong Electronic Technology Co., LTD, and the origin is Shenzhen, China. Subsequently, the acquired image of 4000x3000 pixels was clipped to 800x600 pixels to improve the operation efficiency, and the middle five images were retained to avoid blurring of florets and spikelets. The cropping method is shown in **Figure 1B**. To ensure the accuracy of the experiment, 4570 wheat images of wheat including different varieties (including TKM33, SN48, JM44, and SN27), flowering periods, planting densities, shooting angles, weather conditions, and light intensities were collected.

### 2.2 YOLOv5 model

The convolution neural network (CNN), as a neural network based on the principle of biological visual neural perception, includes convolution computations and a kind of feedforward neural network with a deep structure (Chen et al., 2021; Li et al., 2021). Object detection methods based on deep learning are developing rapidly and can be roughly divided into two types: the two-stage detection method based on candidate region and the one-stage detection method based on regression (Huang et al., 2021; Yun et al., 2022). Among them, the YOLO (You Only Look Once) series was an essential part of one-stage detectors (Redmon et al., 2016). In the task of target detection, YOLO could obtain global-context information by looking at the input images only once.

YOLOv5 is one of the YOLO series models, which achieves a better balance between accuracy and speed than the previous version. The YOLOv5 framework is composed of input, feature extraction module (Backbone), feature fusion module (Neck), and output (Prediction). The backbone network is responsible for feature extraction targets, and the Neck network generates feature pyramids for object scaling. The prediction network adopts three scales of head: small (80×80×128), medium (40×40×256), and large (20×20×512) for final detection. YOLOv5 is divided into YOLOv5n, YOLOv5s, YOLOv5m, YOLOv5l, and YOLOv5x models according to the depth and width of the network. The overall structure of these five models is the same. The width of the network determines the number of convolution kernels, which is the learning ability of the network to extract features. The depth of the network determines the number of components at each level, that is, the ability to fuse features and the speed of model convergence. Considering that this paper is applied to the detection of the wheat flowering period in the field with high real-time requirements, a network model based on improved YOLOv5s is proposed.

### 2.3 Determination method of flowering period based on improved YOLOv5s

**Figure 2:**
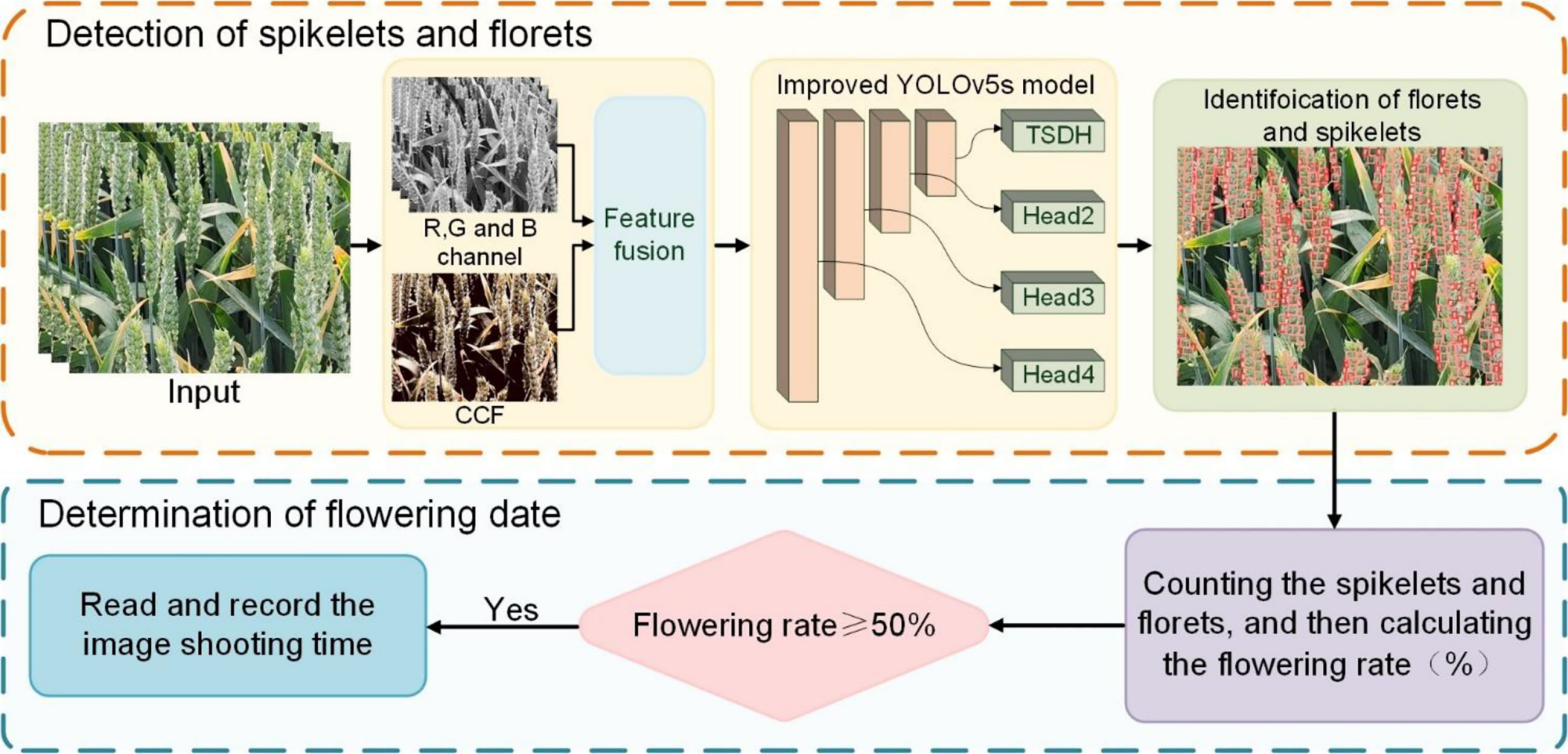
Determination process of wheat flowering period.

The determination method of the wheat flowering period is mainly divided into two steps. The first step is to accurately identify florets and spikelets based on the improved YOLOv5s model. Then the ratio of florets to spikelets was used as the flowering rate. When the flowering rate was over 50%, the time of image capture was read and recorded to complete the determination of the flowering period. The determination process of the flowering period is shown in **Figure 2**.

There are three major improvements from the original YOLOv5s to the improved model are described below: (1) A feature fusion (FF) method that combining RGB images with corresponding comprehensive color features (CCF) was proposed, attenuating the distortion of the extracted feature image caused by light and highlighting more texture features. (2) The convolutional block attention module (CBAM) (Woo et al., 2018) was inserted into the original YOLOv5s network. The CBAM assigned a large weight to floret and spikelet features by combining the channel attention module and spatial attention module. It can learn target features well and suppress non-targets features to improve detection accuracy. (3) Considering that the target scale changed dramatically and a large number of small targets in the wheat dataset, an integrated Transformer small-target detection head (TSDH) was added to combine with the other three detection headers of the original YOLOv5s network for accurate spikelet and floret detection.

#### 2.3.1 Feature fusion method

Compared with rice, corn, and other crops, the background of wheat population images in the field is more complex, the morphological structure of florets and spikelets is small, and the color difference between them is also not palpable (Bommert et al., 2005; Feng et al., 2022; Huang et al., 2022). Converting RGB images to common color spaces such as HSV or Lab does not solve the global noise caused by light, wheat awns, leaves, and soil. Therefore, in the previous study, a comprehensive color feature (CCF) method that can be adjusted adaptively according to the light intensity and clarity of images was proposed by comparing the data feature of different color spaces and different color indices (Liu et al., 2022). In this study, the adaptive adjustment CCF method was used to reduce the influence of light and enhance the differences of florets, spikelets and other targets.

**Figure 3:**
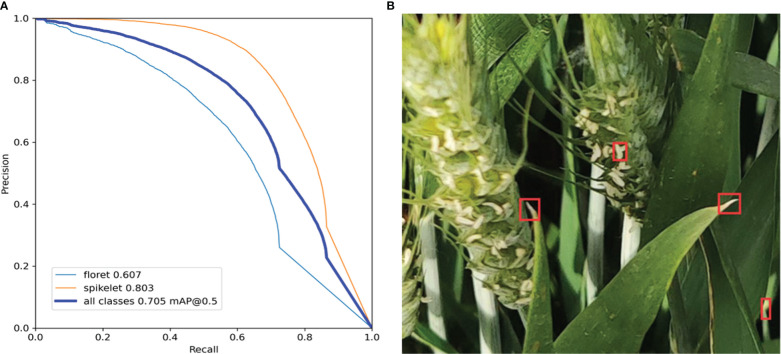
Identify situation of the Original YOLOv5s model. **(A)** precision-recall curve of florets and spikelets(floret 0.607 mAP@0.5, spikelet 0.803 mAP@0.5). **(B)** misidentification of florets(red box).

As shown in **Figure 3A**, the accuracies of the original YOLOv5s model in identifying florets and spikelets were only 60.7% and 80.3%. The detection results of the original YOLOv5s model showed that the main reason for the low recognition accuracy is a relatively uniform degree of feature standard extracted from RGB images. However, different wheat varieties have different traits, and there is a lack of high-weight added-value eigenvectors that can highlight the characteristics of florets and spikelets in the process of model classification. Due to the small floret target and high noise in the field environment, the original YOLOv5s model would greatly increase the probability of missing recognition and misidentification when raising the detection threshold. This made a huge difference between precision and recall. The characteristics of florets and leaf reflective spots are similar under natural conditions, which led to misrecognition and the reduced detection ability of the original YOLOv5s model for florets. As shown in **Figure 3B**, wheat leaves were mistaken for florets. Therefore, more dominant traits need to be provided decision support for the detection layer.

**Figure 4:**
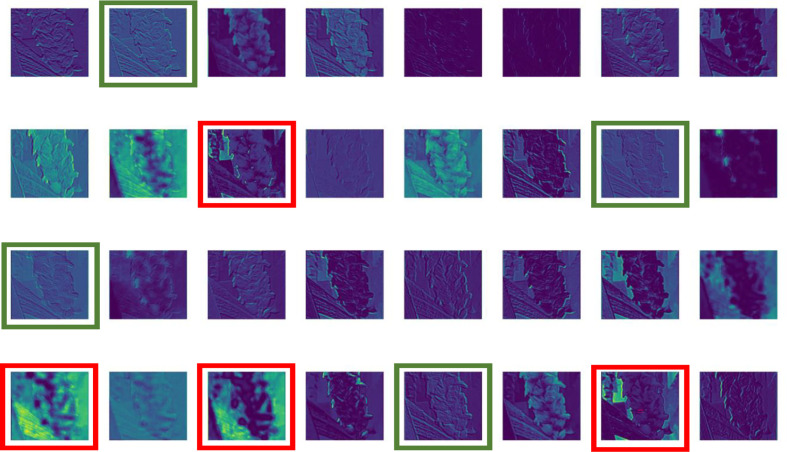
Partial feature map extracted by the Original YOLOv5s model (Red marked box: distortion features. Green marked box: redundant features).

**Figure 4** shows that both shallow and deep features after convolutional extraction have distortion features (red marked box) caused by the light and redundant features (green marked box) that increase model convergence time during training. To solve the above problems, the main methods available are to reduce the number of convolution cores or to fuse images in multiple sources to increase the extraction of effective features. However, the former is suitable for situations where the background of image data is highly controllable, such as indoor environment or image data collected through a standardization process, but not for complex field environments (Liu et al., 2021; Zhao et al., 2021; Bai et al., 2022).

**Figure 5:**
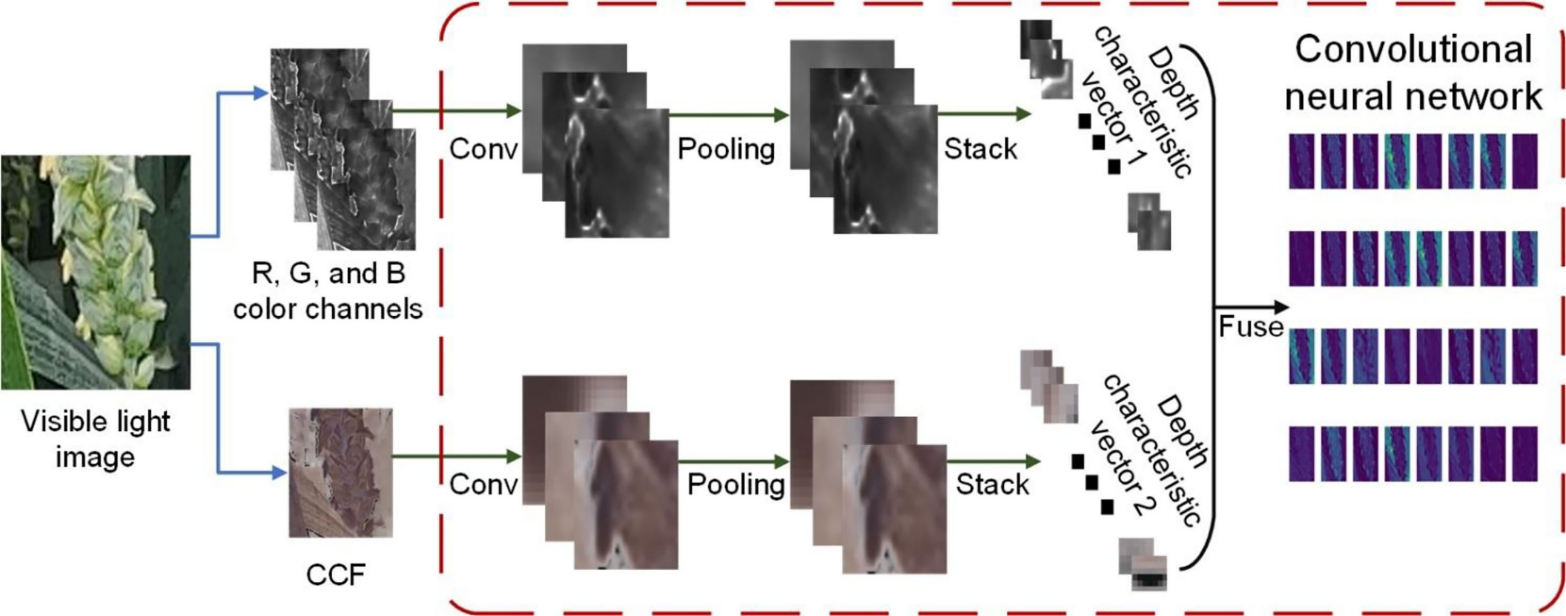
The overall schematic diagram of feature fusion method.

Therefore, a feature fusion (FF) method combing RGB images and corresponding CCF was presented to solve the above difficulties, and subsequent feature extraction and feature fusion are performed based on the fused images. The feature fusion method workflow, illustrated schematically in **Figure 5**, mainly contains three steps: (1) The R, G, and B channels in RGB images and the channel information of the CCF are extracted and transmitted to the input layer of the convolutional neural network. (2) In the feature extraction stage of the YOLOv5 model, the shallow and deep features of the two image sources input in step 1 are extracted. (3) The input features are addressed by independent convolution, pooling, and full connection networks, and the extracted features are stitched with equal weight.

In **Figure 5**, the main function of the convolution layer is to extract local features from the pixel information of input images. The scale of features extracted by the convolution kernel is relatively large, and using the above features for target classification will produce a large amount of computation and affect the inference speed. Therefore, secondary extraction of image features using a pooling layer reduces the feature parameters. Considering that the background of the field image is complex and noisy, pooling the extracted features with kernel sizes of 3, 5, and 7 to better preserve the feature texture and improve the generalization of the model. The same gradient descent algorithm as pre-training was used for model training, and the parameters of the convolution layer and pooling layer in the convolutional neural network were updated by back propagation.

**Figure 6:**
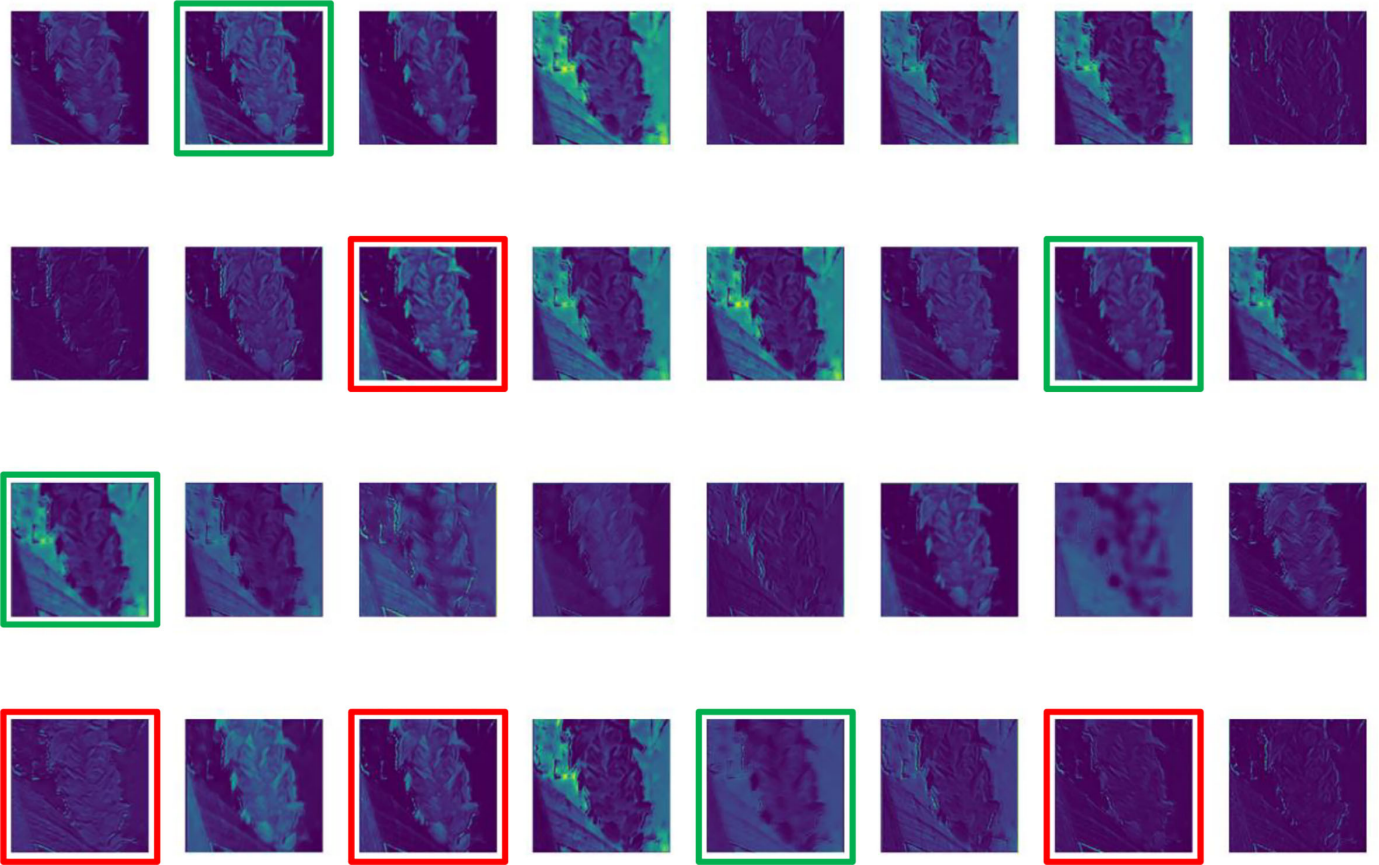
Partial feature map extracted based on feature fusion method.

Compared with **Figure 4**, the proposed feature fusion method attenuated the distortion of the extracted feature image caused by light and highlights more texture features. It is likewise easier to classify florets and spikelets in shallow features with clearer details, and the results are shown in **Figure 6**.

#### 2.3.2 Convolutional block attention module

The complementarity between RGB images and corresponding CCF was enhanced by the feature fusion method in the previous section. The feature fusion method eliminated the influence of background noise and extracted more characteristics of florets and spikelets in complex environments, but increased the input image from three to four dimension. The attention mechanism was integrated into the YOLOv5s model to improve the model, could make the network more focused on spikelets and florets, and avoid too many features affecting the computational power and convergence speed of the model. The Convolutional block attention module (CBAM) is one of the most effective attention mechanisms and consists of channel attention module and spatial attention module. It can enhance the ability of the model to extract image features and suppress invalid background information by redistributing the originally uniform distribution resources according to the importance of the detection target. So, the CBAM was adopted into the feature fusion layer of YOLOV5s model. The calculation process of the CBAM is shown in Equation (1).


(1) $$\begin{array}{l} F^{\prime }=M_{C}\left(F\right)⊗F\\ F^{\prime \prime }=M_{S}\left(F^{\prime }\right)⊗F^{\prime } \end{array}$$

In the above formula, *F* represents the input eigenvector, *MC* is the channel attention feature map, *F* ^′^ represents the channel attention module outputs feature vectors, *MS* is the spatial attention feature map, and *F* ^″^ represents the spatial attention module outputs feature vectors.

**Figure 7:**
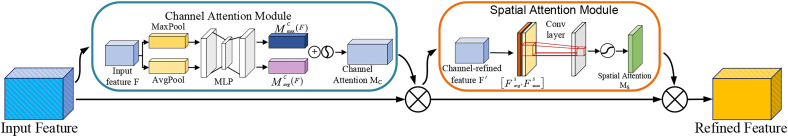
The overall structure of convolutional block attention module: MaxPool represents the maximum global pooling; AvgPool represents global average pooling; MLP represents a multi-layer perceptron with shared weights; Conv indicates convolution operation.

CBAM links the channel attention module and spatial attention module in series. The channel attention module first compressed the input feature map by average pooling and maximum pooling, and then sent it to the shared multi-layer perceptron (MLP) structure for processing. Finally, the channel attention map was generated by the sigmoid function to solve the problem of what is the target. The spatial attention module used convolution and the sigmoid function to process the input feature map and ultimately determined where to pay attention. Compared with separate channel attention network SENet and spatial attention network STN, the CBAM does not increase too much computation (Zhao et al., 2022). It is a lightweight module that can be integrated into the most well-known CNN architecture and can be trained in an end-to-end manner. The CBAM only needs to give a feature mapping at the convolution layer, then it will infer attention mapping in turn along two independent dimensions, channel, and space. The attention map is then multiplied by the input feature to perform adaptive feature refinement. Therefore, the CBAM is a simple but efficient attention module, and its module structure is shown in **Figure 7**.

The CBAM was added after the C3 module of the neck to update feature mapping weights after each residual convolution in the feature fusion stage. It can effectively improve the accuracy of the improved YOLOv5s in florets and spikelets target detection by weighting the feature map weights of different channels and spatial dimensions.

#### 2.3.3 Multi-detection heads structure integrated transformer

**Figure 8:**
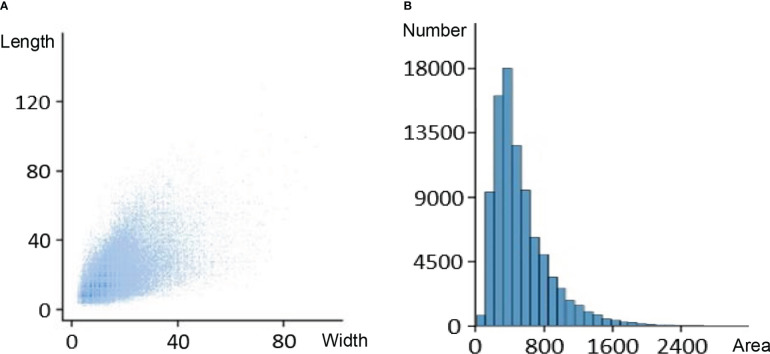
Labeled data distribution: **(A)** size distribution. **(B)** area distribution.

After normalizing the coordinate dimensions in the acquired dataset, the length and width size distributions and pixel area distribution of each labeled target were collected for statistics. The result is shown in **Figure 8**. Without data enhancement, the pixel area of 9325 labeled boxes is less than the minimum detection box size of YOLOv5s, accounting for about 10% of all labeled boxes.

It was found that the whole image of the wheat field contained many tiny examples due to the small size of florets and spikelets analyzing the labeled data. So, an up-sampling operation was inserted into the neck of YOLOv5s and a detection head was embedded to detect tiny targets. The improved microscale layer generated a feature map by extracting the underlying spatial features and fusing them with deep semantic features, which made the network structure more extensive and detailed. Combined with the other three detection heads, the improved YOLOv5s four-head structure can alleviate the negative effects caused by the large size distortion of florets and spikelets.

**Figure 9:**
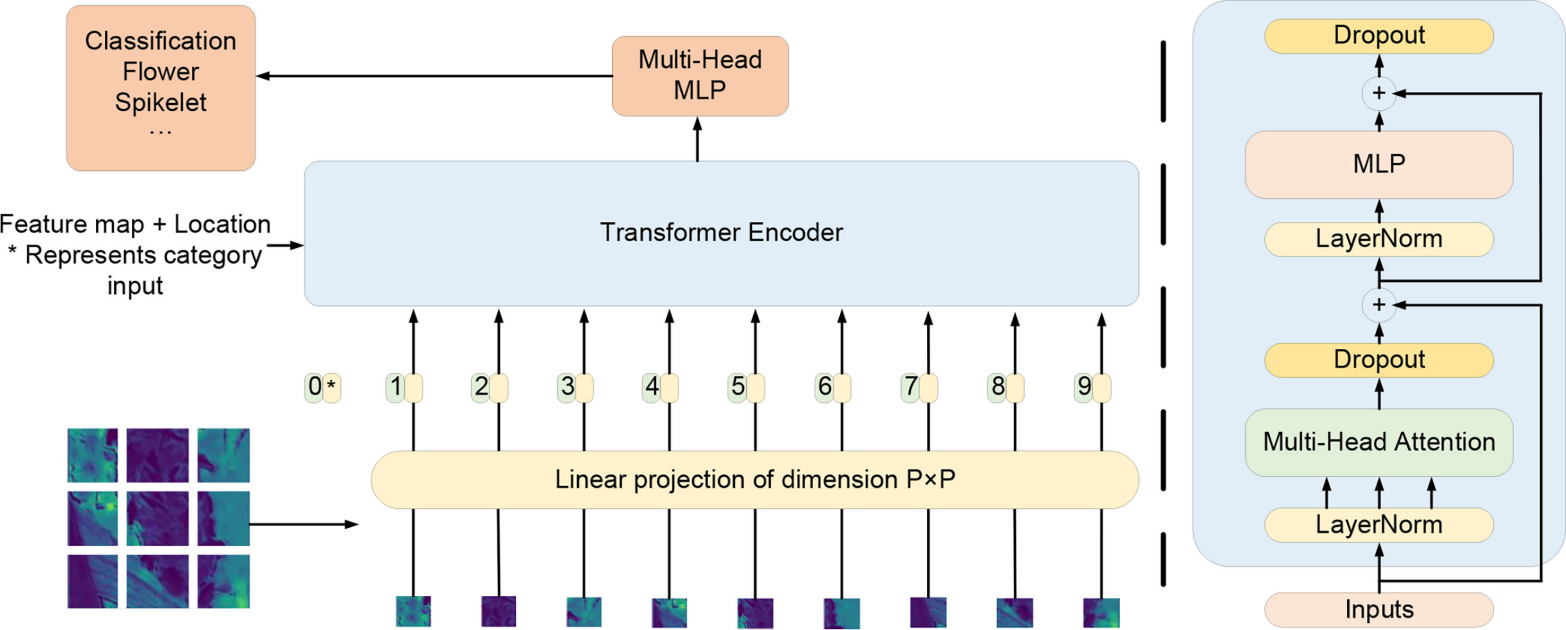
The diagram of transformer encoder structure.

The newly added detection head is located at the end of the neck of YOLOv5s. After multiple up-sampling, the resolution of the feature map is lower, and blurred small targets will be missed. Inspired by Vision Transformer (Dosovitskiy et al., 2020), the C3 module originally connected to the newly added detection head was replaced with the Transformer encoder module shown in **Figure 9**. In the first step, the feature map with the input scale of W×H was divided into 1 × 1 pixel blocks, and the positions of each pixel block in the feature image were sequentially entered into the encoder. Transformer encoders are composed of two sub-layers. The first is the multi-head attention layer, and the second (MLP) is the fully-connected layer. Each sub-layer is connected using residual connections. To improve model generalization and reduce computation costs, the dropout layer is behind each sublayer. Different eigenvectors in the encoder containing spatial location information were transferred to the dropout layer through the attention module of the feed-forward neural network. The dropout layer discarded the low-weight features and then transmitted the valid features to the full connection layer to complete the classification task.

**Figure 10:**
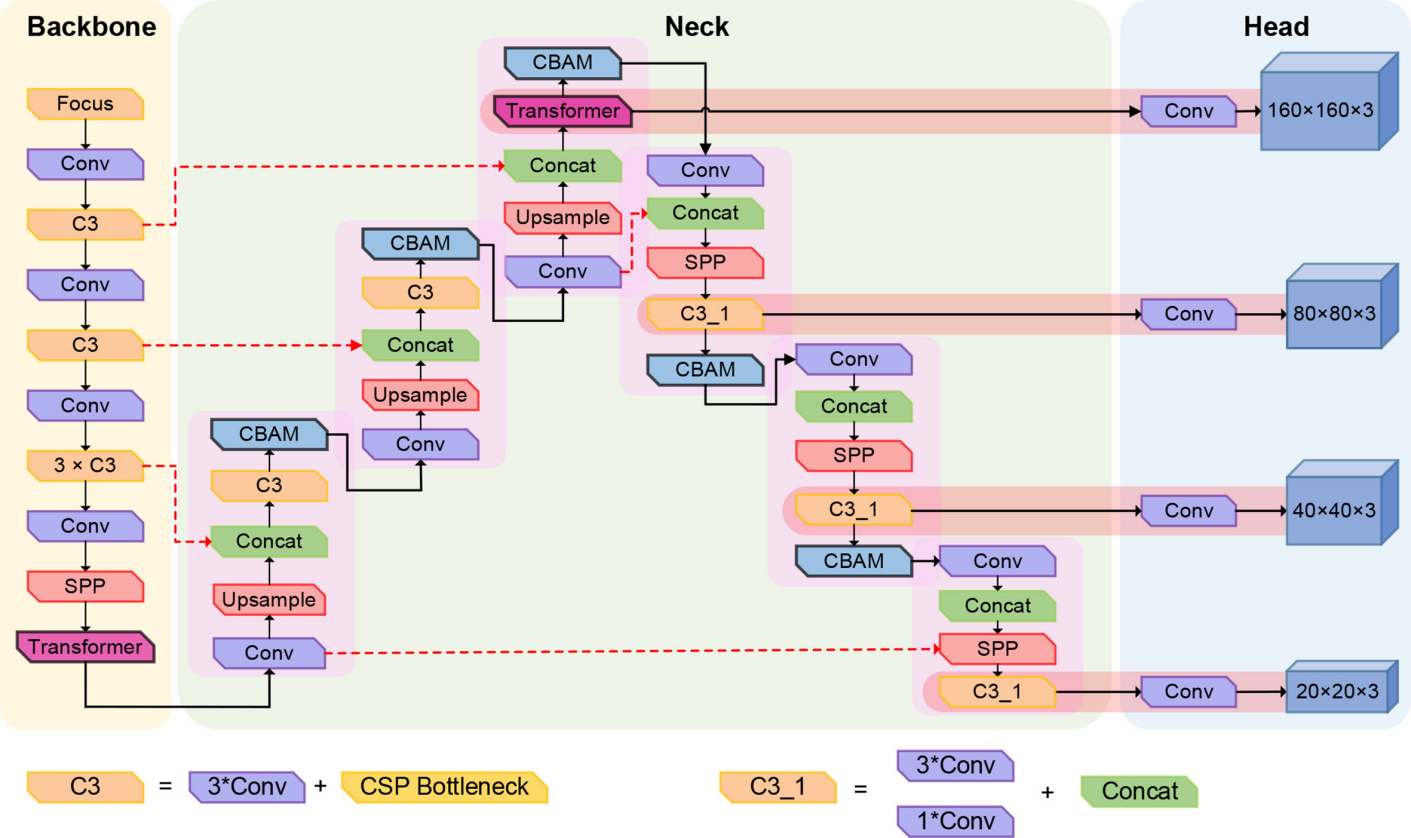
Schematic diagram of improved YOLOv5s network structure.

The ability to capture global information and upper-level features can be enhanced by integrating Transformer small-target detection header. Furthermore, it can exploit the self-attention mechanism to explore the potential of feature representation (Zhu et al., 2021). The overall structure of the improved network is shown in **Figure 10**.

Multiple convolution and up-sampling operations are required while adding a detection head. To avoid slow model convergence and gradient explosion caused by the increase of feature dimensions, the maximum pooling layer (SPP) was embedded in front of the second, third, and fourth detection heads. The added maximum pooling layer can ensure the integrity of the overall image features while reducing parameters. **Table 1** presents the parameters of the improved YOLOv5s model.

**Table 1:** Improved layer parameters of YOLOv5s network.

Layer	Module	stack	Output size	Source	Input dimension	Output dimension	Convolution
0	Focus	1	320	-1	3	32	3×3
1	Conv	1	160	-1	32	64	3×3
2	C3	1	160	-1	64	64	[1, 3]
3	Conv	1	80	-1	64	128	3×3
4	C3	1	80	-1	128	128	[1, 3]
5	Conv	1	40	-1	128	256	3×3
6	C3	3	40	-1	256	256	[1, 3]
7	Conv	1	20	-1	256	512	3×3
8	SPP	1	20	-1	512	512	[3, 5, 7]
9	Trans	1	20	-1	512	512	—
10	Conv	1	20	-1	512	256	1×1
11	Upsample	1	40	-1	256	256	—
12	Concat	1	40	-1, 6	256	512	—
13	C3	1	40	-1	512	256	[1, 3]
14	CBAM	1	40	-1	256	256	—
15	Conv	1	40	-1	256	128	1×1
16	Upsample	1	80	-1	128	128	—
17	Concat	1	80	-1, 4	128	256	—
18	C3	1	80	-1	256	128	[1, 3]
19	CBAM	1	80	-1	128	128	—
20	Conv	1	80	-1	128	64	1×1
21	Upsample	1	160	-1	64	64	—
22	Concat	1	160	-1, 2	64	128	—
23	Trans	1	160	-1	128	128	—
24	CBAM	1	160	-1	128	128	—
25	Conv	1	80	-1	128	64	3×3
26	Concat	1	80	-1, 20	64	128	—
27	SPP	1	80	-1	128	128	[3, 5, 7]
28	C3_1	1	80	-1	128	256	[1, 3]
29	CBAM	1	80	-1	256	256	—
30	Conv	1	40	-1	256	128	3×3
31	Concat	1	40	-1, 15	128	256	—
32	SPP	1	40	-1	256	256	[3, 5, 7]
33	C3_1	1	40	-1	256	512	[1, 3]
34	CBAM	1	40	-1	512	512	—
35	Conv	1	20	-1	512	256	3×3
36	Concat	1	20	-1, 10	256	512	—
37	SPP	1	20	-1	512	512	[3, 5, 7]
38	C3_1	1	20	-1	512	1024	[1, 3]
39	Detect	1	20,40,60,120	23,28,33,38	—	—	—

#### 2.3.4 Determination of flowering period

The actual flowering period could not be accurately determined by the ratio of the flowering spikes to the no-flowering spikes. Because one flower or ten flowers on a single wheat spike can be counted as a flowering spike, the true flowering condition of the single wheat spike cannot be fully judged, thus affecting the determination of the wheat flowering period. The agronomic criterion for the current wheat flowering period identification is that the inner and outer glumes of the florets in half of the spikelets in the plot are opened and the pollen is dispersed. At present, the flowering period of a large number of wheat breeding materials is mainly determined by manual estimation of the proportion of florets to spikelets (Zhang et al., 2020; Wang et al., 2020). So this study determined the flowering period of a plot based on the flowering rate, which is the ratio of the spikelets to florets in all images obtained by the community. When the flowering rate exceeded 50%, the plot was determined to be in flowering period, otherwise, it was determined to be heading period.

### 2.5 Experimental training and evaluation indicators

The dataset in this paper is composed of spikelets and florets, and the field environment is relatively complex. The effect will be better if there are public datasets with similar detection tasks to use the transfer learning training model. However, no similar detection task dataset was found at present, so transfer learning was not performed. For a fair comparison between the models, each model was trained from scratch. The learning rate was based on cosine annealing attenuation strategy (Loshchilov and Hutter, 2016), as shown in Equation (2), and the number of iterations was 300.


(2) $$n_{t}=n_{\min}^{i}+\frac{1}{2}\left(n_{\max}^{i}-n_{\min}^{i}\right)\left(1+\cos\left(\frac{T_{i}}{T}\Pi \right)\right)$$

In the above formula, $n_{\min}^{i}$ , $n_{\max}^{i}$ represent the minimum and maximum of the *i*-round learning rate, *T*_*i*_ represents the cumulative number of samples during the *i*-round training, and *T* represents the total number of samples.

The comprehensive recognition accuracy and real-time performance of the model under the five categories of florets, spikelets, and background were measured using three indicators: precision *P*, recall rate *R*, *F1-score*, *accuracy*, and mean average precision *mAP*, as shown in Equations (3-7).


(3) $$P=\frac{TP}{TP+FP}$$

(4) $$R=\frac{TP}{TP+FN}$$

(5) $$F1-score=\frac{2PR}{P+R}$$

(6) $$mAP=\frac{1}{c}\sum_{k=i}^{N}P_{\left(k\right)}R_{\left(k\right)}$$

(7) $$Accuracy=\frac{TP+TN}{TP+FN+FP+TN}$$

Among them, *TP* represents the number of spikelets and florets correctly identified by the model, *FP* represents the number of false recognition of the background as spikelets and florets, *FN* represents the number of spikelets and florets not identified, *TN* represents the number of background correctly identified by the model, *C* represents the sample category, *N* represents the threshold of citations.

The higher the value of *P*, *R*, and *mAP*, the higher the accuracy of object detection, and the average run time (ms/frame) was the average time the model takes to process the wheat image.

## 3 Results and discussion

The florets identification and the determination of the flowering period of wheat populations in the field have yet to be studied. To verify the effectiveness and adaptability of the improved YOLOv5s model under different conditions, quantitative and qualitative tests were conducted on the improved YOLOv5s model and the original YOLOv5s model in the test set images. And the performance differences between the improved model and other models were studied by comparing with other advanced and non-deep learning methods. Afterward, an ablation experiment was conducted to explore the optimization effect of various improvement strategies applied in the YOLOv5s model, enabling better detection capability in this work. Finally, the actual performance of the model for flowering determination of wheat population in the field was further tested, and the field phenotype acquisition platform was invoked as the carrier to conduct field experiments.

### 3.1 Quantitative test

**Figure 11:**
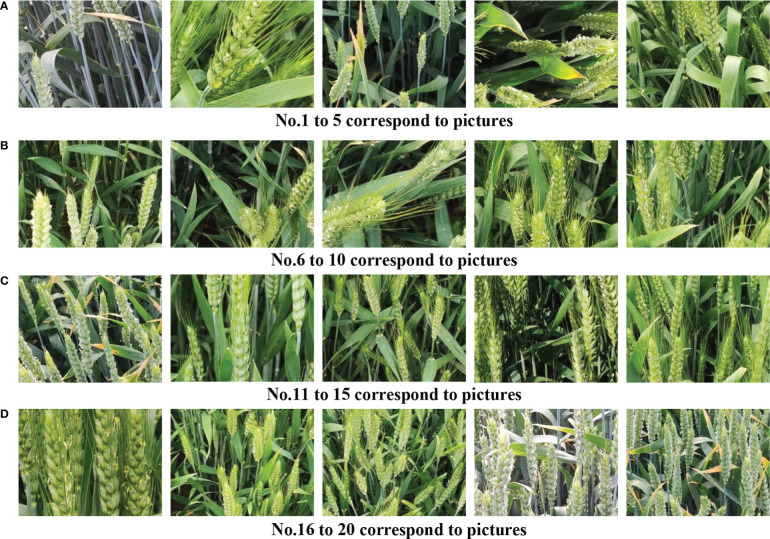
Twenty images with different numbers of spikes, different varieties, different shooting angles and planting densities. **(A)** The numbers 1 to 5 correspond to the picture. **(B)** The numbers 6 to 10 correspond to the picture. **(C)** The numbers 11 to 15 correspond to the picture. **(D)** The numbers 16 to 20 correspond to the picture.

From 457 images in the test set, 20 images with different panicles, different varieties, shooting angles, and different planting densities were selected for calculation and analysis as shown in **Figure 11**. Florets and spikelets were counted by manual method and model respectively, and the classification accuracy was calculated. The statistical results are shown in **Table 2**. The confidence threshold was placed at 50%. The original YOLOv5s model is represented by model 1, and the improved YOLOv5s model is represented by model 2.

**Table 2:** Counting statistics results.

No.	Number of florets			Number of spikelets		
	Manual count	Model 1	Model 2	Manual count	Model 1	Model 2
1	48	34	39	57	50	54
2	7	5	5	46	40	43
3	69	38	47	121	94	106
4	52	31	43	107	86	99
5	15	12	13	94	88	98
6	43	28	36	129	117	128
7	38	24	35	50	39	45
8	32	22	29	44	34	40
9	80	42	73	86	73	79
10	52	38	44	110	95	107
11	139	87	103	226	192	211
12	16	11	11	62	55	59
13	36	21	27	134	118	132
14	38	22	33	115	98	112
15	81	54	72	148	117	137
16	52	37	48	68	66	66
17	58	31	42	216	173	190
18	91	67	78	273	237	256
19	146	95	121	214	159	191
20	188	131	155	335	276	309

(Model 1 represents the original YOLOv5s model. Model2 represents the improved YOLOv5s model.).

**Table 2** shows that the improved YOLOv5s model and the original YOLOv5s model have high recognition accuracy in images with low planting density and low background noise (such as No. 2, 5, 12, and 16). Comparing images No. 4, 7, and 8, when the degree of occlusion is small and the target is obvious, the original YOLOv5s model has poor adaptability to the distortion of wheat morphology. The improved YOLOv5s model has higher detection accuracy for distorted florets and spikelets, and the recognition accuracy rate was 87.7% and 91.5%. When the influence of light is strong (such as images No. 6, 14, and 19), the recognition accuracy of the original model for florets and spikelets was only 63.9% and 81.2%, and the recognition accuracy of the improved model was 83.7% and 94.1%. For wheat images with large differences in characters (such as images No. 1, 11, 17, 18, and 20), compared with the original YOLOv5s model, the recognition accuracy of florets and spikelets by the improved YOLOv5s model was improved by 12.8% and 8.3% respectively.

For a more intuitive reflect the recognition effect of the model, the evaluation criteria for 457 images in the test set are shown in **Table 3**. The improved YOLOv5s model has a detection precision of 95.3%, recall rate of 86.2%, *mAP* of 92.9%, and an average detection time of 11.5ms for a single image. Although the detection time was increased by 38.8% compared with the original YOLOv5s model, the number of video frames detected per second was 86Fps, which is suitable for the current mainstream visible light cameras.

**Table 3:** Detection results of improved YOLOv5s model test on the test dataset.

Models	Recognition accuracy	Running time/s
YOLOv5s	*P* = 0.719 *R* = 0.637 *mAP* = 0.705	3.79
**Improved YOLOv5s**	*P* = 0.953 *R* = 0.862 *mAP* = 0.929	5.26

### 3.2 Qualitative tests

**Figure 12:**
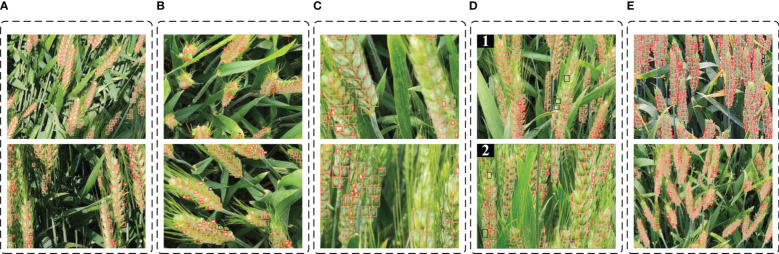
Detection effect of improved YOLOv5s model in different complex situations (The pink and dark red boxes represent true positive; black boxes represent false negative; blue boxes represent false positive). **(A)** strong light. **(B)** angle distortion. **(C)** blurred target area. **(D)** severe occlusion. **(E)** population phenotype enrichment.

The effectiveness and adaptability of the improved YOLOv5s model under various conditions were proved by quantitative experiments. In order to test the recognition effect of the model in various situations more intuitively, some images were selected for qualitative tests. As shown in **Figure 12**, this section divides some photos in the test set into five categories for qualitative tests: strong influence of light effect, severe angular distortion, blurred target area, serious occlusion, and population phenotype enrichment. All of the above are important factors affecting the robustness of the model in field image detection (Hu et al., 2019; Ajlouni et al., 2020).

Under the influence of strong light, the surface reflection of spikelets is serious, and the visual effect at the texture and endpoint of the leaves is similar to the color characteristics of the floret, which can easily cause misrecognition. The recognition situation when the local or global light is strongly affected is shown in **Figure 12A**. Only once had the leaf been misidentified as the spikelet, as indicated by the blue box in the figure. It can be seen from the recognition effect that the improved model had good adaptability to light, and there was no missing detection of wheat spikes and false detection of florets due to light reflection.

Wheat at the flowering period just shifts from vegetative growth to reproductive growth, the stem is soft and the accumulation of dry matter in the spike increases, causing the wheat to show signs of lodging. The angle or shape distortion of the collected images caused by the skew of the spikelet is shown in **Figure 12B**. According to the results, the improved YOLOv5s model had a beneficial effect on the detection of floret targets under distortion. However, when the tilt angle of spikelets is large, the spikelets are too dense, leading to a small number of spikelet targets with large distortion would be missed. The black box in the figure shows missing spikelets.

Phenotypic acquisition platform with large bumps and undulations during driving, as well as excessive wind speed during photographing, would cause blurring of the collected image information. Shown in **Figure 12C** is the recognition effect of fuzzy targets, in which the model prediction performance was better for distant blurred targets but less effective for near targets with larger target sizes. By detecting the fuzzy images in the test set, the recognition accuracy of florets and spikelets was 86.4% and 90.7%, respectively, reduced by 2.5% and 6.1% compared with the average recognition accuracy of the improved model. The improved YOLOv5s model had no significant reduction in recognition accuracy due to image blurring and has good adaptability to target blurring.

Different wheat varieties have distinct traits. It was noted that some varieties of wheat had long and dense awns, which was the main factor to produce image noise by comparing different wheat varieties. Some wheat varieties had a serious overlap of spikes due to the high number of tillers. The recognition of serious occlusion overlaps is shown in **Figure 12D**. As shown in **Figure 12D**-2, the improved YOLOv5s model had higher accuracy in identifying the wheat varieties with severe overlap. However, in **Figure 12D**-1, the noise produced by over-dense wheat awns of this variety caused the hidden florets and spikelets to be missed detection. The black box in the figure shows the missed detection of florets and spikelets.

In the plots with high planting density, the acquired images contain rich phenotypic information of the population, as shown in **Figure 12E**. The area of pixels occupied by the region of interest would tend to be less and is prone to small target missing when the image contains too much phenotypic information. The improved YOLOv5s model can accurately determine the region of interest and detect florets as well as spikelets, as seen by the identification effect.

### 3.3 Comparative experiments of different object detection algorithms

This study was compared with other advanced deep learning and non-deep learning methods to investigate the performance differences between the improved YOLOv5s model and other models. To guarantee the reliability of the results, the six networks(i.e., YOLOv3, YOLOv7, Faster R-CNN, Cascade R-CNN, superpixel segmentation, Improved YOLOv5s) were trained using the training and validation datasets in the same training environment. The training results are shown in **Table 4**, and the improved YOLOv5s achieved the best performance in all indicators, including the accuracy of florets and spikelets was 88.9% and 96.8%, F1-score of 90.52%, and mean average precision of 92.9%. Taken together, the improved YOLOv5s method presented had the best performance for detecting florets and spikelets compared with other detection methods, which proved the validity of the model proposed in this paper.

**Table 4:** Indicators results of the six models on the test set.

Test indicators	Accuracy of spikelets (%)	Accuracy of florets (%)	F1-score(%)	mAP_0.5 (%)
YOLOv3	61.0	28.0	54.0	45.5
YOLOv7	82.4	62.2	71.5	72.5
Faster R-CNN	76.8	71	67.2	57.3
Cascade R-CNN	71.0	57.0	66.4	56.7
Based on CCF and superpixel segmentation	76.3	70.6	/	/
**Improved YOLOv5s**	**96.8**	**88.9**	**90.5**	**92.9**

### 3.4 Ablation studies

In the previous section, quantitative and qualitative tests were carried out for the detection of the improved YOLOv5s model under various complex conditions and a quantitative comparison was made with the detection result of the original YOLOv5s model in the test set. The validity and adaptability of the improved model were proved. As mentioned earlier, three major improvements were made to the original YOLOv5s, including image enhancement (e.g., feature fusion of RGB images with the corresponding CCF; described in subsection 2.3.1) and some structural changes to the network (e.g., adding the Convolution block attention module, and the integrated Transformer small-target detection head; described in subsections 2.3.2 and 2.3.3). Therefore, an ablation experiment was conducted on the improved YOLOv5s model to explore the contribution of the proposed improvement strategies to the model detection performance improvement. The corresponding detection indicators for each optimization strategy are presented in **Table 5**.

**Table 5:** Detection indexes of the model with different optimization strategies.

	Flowering detection of wheat in field			
Feature fusion	×	√	√	√
Convolutional block attention model	×	×	√	√
Multi-detection heads structure integrated transformer	×	×	×	√
*P* (%)	71.9	84.5	90.0	**95.3**
*R* (%)	63.7	71.0	77.6	**86.2**
*F1-score* (%)	67.6	77.2	83.3	**90.5**
*mAP_0.5* (%)	70.5	78.8	86.9	**92.9**

**Figure 13:**
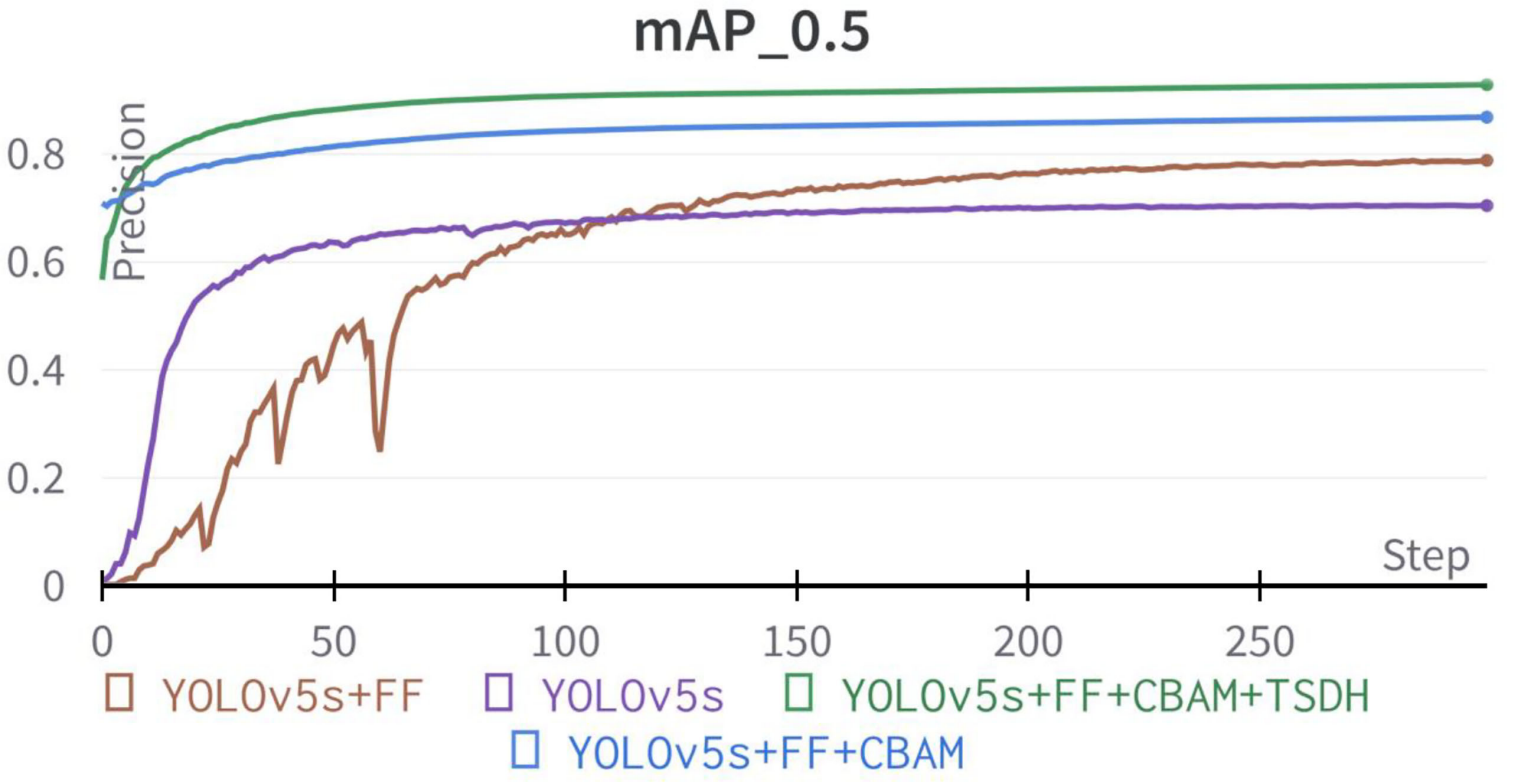
The mean average precision comparison of YOLOv5s prediction model based on different improvement strategies.

As shown in **Table 5**, the improved YOLOv5s model proposed greatly enhanced various metrics of flowering detection in field wheat relative to the original model. The precision of the original YOLOv5s model was 71.9%, recall rate of 63.7%, F1-score of 67.6%, and mAP at 50% confidence of 70.5%. The P, R, F1-score, and mAP of the improved YOLOv5s model were increased by 23.4%, 22.5%, 22.9%, and 22.4%, respectively compared with the original YOLOv5s model. The *mAP* comparison test of the YOLOv5s prediction model based on different improvement strategies is shown in **Figure 13**.

**Figure 14:**
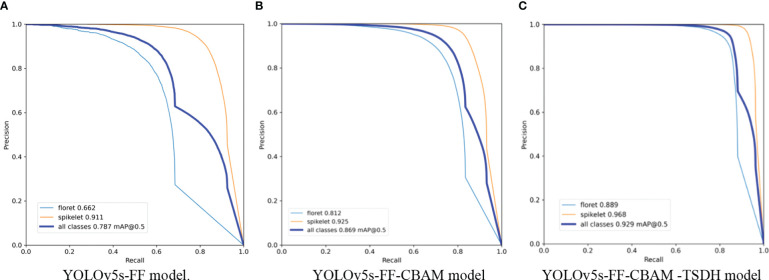
The precision-recall curves of florets and spikelets by different models. **(A)** precision-recall curve of YOLOv5s-FF model(floret 0.662 mAP@0.5, spikelet 0.911 mAP@0.5). **(B)** precision-recall curve of YOLOv5s-FF-CBAM model(floret 0.812 mAP@0.5, spikelet 0.925 mAP@0.5). **(A)** precision-recall curve of YOLOv5s-FF-CBAM-TSDH model (floret 0.889 mAP@0.5, spikelet 0.968 mAP@0.5).

Among the three optimization strategies, the detection model with the addition of feature fusion method improved the *P*, *R*, *F1-score*, and *mAP* by 12.6%, 7.3%, 9.6%, and 8.3%, respectively, compared with the original model. The effect of the detection model integrating the feature fusion method is shown in **Figure 14A**. It can be observed in the comparative test that the improved feature fusion method greatly improves the accuracy of the model, among which the recognition accuracy of florets and spikelets was increased by 5.5% and 10.8%, respectively. This is mainly because the feature fusion method reflects more texture characteristics of the wheat field images. As the target of florets is smaller than spikelets and the image background in RGB images is more complex, making it is difficult to extract the morphological features of florets. In addition, light points at the tip of spikelets and leaves could also be misidentified as florets under the influence of light, resulting in weak floret detection performance of the original YOLOv5s model. Thus, the performance of model detection was greatly improved by combining the feature fusion method.

The channel attention module and space attention module were introduced on the basis of the feature fusion method, which also improved the detection effect of the model. The precision, recall rate, *F1-score*, and *mAP* of the improved model were improved by 5.5%, 6.6%, 6.1%, and 8.1% respectively in the validation set. Experimental results show that the performance of the YOLOv5s model embedded with the convolutional block attention module was greatly improved.

As presented in **Figure 14B** the detection accuracy of florets and spikelets increased by 15.0% and 1.4% respectively, proving the effectiveness of the improvement. High-density planting and characteristic mapping of different traits are the main reasons for model performance degradation, on complex field images. After embedding the CBAM, the influence of occlusion and noise on the model can be weakened by assigning weights to different feature maps, and the useful target objects can be focused. Therefore, the improvement effect was significant.

The range of receptive fields obtained by different size detection heads also varies greatly, which reflects the ratio of feature maps to the input image area. When the receptive field is small, the number of elements in the original image is also small, thereby weakening the detection effect of larger targets. Conversely, when the receptive field is too large, the fine-grained information such as the spatial structure of small targets will be lost, leading to poor recognition effect of distant targets. In order to improve the detection performance of the model for small targets, the transformer detection head structure was added for tiny object detection. Combined with the other original three detection heads of YOLOv5s, the four-head structure can alleviate the negative influence caused by drastic changes in object size. After adding the integrated Transformer small-target detection head, the smallest detection box contained image size of 4x4 pixel, which solved the problem of high detection rate of small targets in wheat group images. The detection head structure added based on the above two types of improvement strategies improved the precision, recall rate, *F1-score*, and *mAP* of the model by 5.3%, 8.6%, 7.2%, and 6.0% respectively. As shown in **Figure 14C**, compared with the P-R curves of the model before adding the integrated Transformer small-target detection head, the improved model improved the recognition accuracy of florets by 7.7% and spikelets by 4.3%. The main improvement point is to optimize the recognition accuracy of the model for long-distance florets and spikelets and solve the problem of missing detection of long-distance tiny objects. After adding the integrated Transformer small-target detection head, although the performance index is less improved, it is essential to realize the accurate identification of florets and spikelets in field population images of wheat.

### 3.5 Comparisons using different attention methods

In this study, CBAM was integrated into the neck of YOLOv5 to improve the model. To evaluate the effectiveness of the CBAM, several state-of-the-art attention methods were applied to the improved YOLOv5s model for comparison. The selected attention methods include Squeeze-and-Excitation Networks(SENet) (Hu et al., 2018), Efficient Channel Attention(ECA-Net) (Wang et al., 2020), Normalization-based Attention Module(NAM) (Liu et al., 2022), Coordinate Attention(CA) (Hou et al., 2021) and Effective Squeeze-Excitation(eSE) (Lee and Park, 2020). The evaluation metrics include *P*, *R*, *F1-score*, and *mAP.* The evaluation performance index results are shown in **Table 6**. Among the five attention mechanisms compared, the improved YOLOv5s model based on CA had the best comprehensive performance, the *P* of 85.2%, *R* of 71.8%, *F1-score* of 77.9%, and *mAP* of 81.4%. The improved model based on CBAM improved the P, R, F1-score, and mAP by 10.1%, 14.4%, 12.6%, and 11.5%, respectively, compared with the CA. The high density of wheat population in the field led to the shielding between wheat spikes, leaves, wheat awn, and stalks. The CBAM started from two scopes, channel and spatial, and allocated attention to two dimensions simultaneously, which enhances the effect of attention mechanism on model performance. The experimental results show that the performance of the improved YOLOv5s model based on CBAM had been greatly improved.

**Table 6:** Comparisons of different attention methods under the improved YOLOv5s.

Model	P (%)	R (%)	F1-score (%)	mAP_0.5 (%)
**YOLOv5s+FF+CBAM+TSDH**	**95.3**	**86.2**	**90.5**	**92.9**
YOLOv5s+FF+SE+TSDH	83.1	71.0	76.6	80.0
YOLOv5s+FF+ESE+TSDH	85.3	71.4	77.7	81.2
YOLOv5s+FF+ECA+TSDH	84.2	71.6	77.2	81.0
YOLOv5s+FF+CA+TSDH	85.2	71.8	77.9	81.4
YOLOv5s+FF+NAM+TSDH	84.6	74.5	77.5	81.0

### 3.6 Flowering period experiment in field

The method proposed in this paper is based on the improved YOLOv5s model to achieve accurate identification of florets and spikelets, and to determine the flowering period of wheat based on the ratio of florets to spikelets. Hence, for the spikes of closed-flowering types, the presented method more likely will not work, because the key morphological features for recognition of florets (anthers) are enclosed. Therefore, the proposed method is suitable for wheat cultivars with open-flowering types of spikes, in which the stamens dangle from the florets.

**Figure 15:**
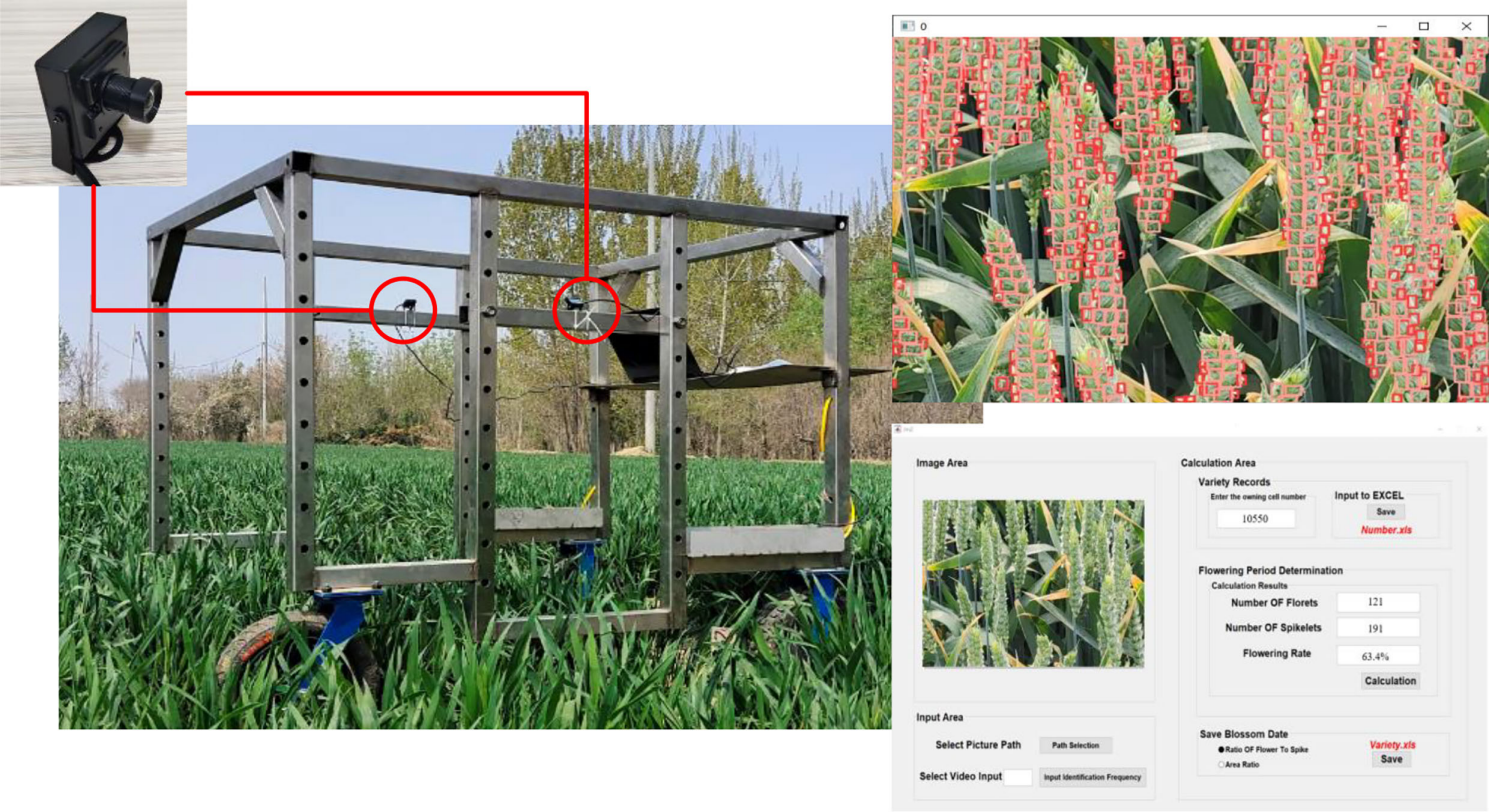
Field experiment process.

Qualitative experiments showed that the improved model had good adaptability to the images of different angles, densities, and distances collected in the field. Since the camera can collect phenotypic information by video or timing shooting, this section will conduct the flowering period experiment in field to detect the image data obtained by different acquisition methods under different flowering conditions. The flowering rate was calculated based on the number of detected florets and spikelets to estimate the overall flowering situation of the plot and determine the flowering period. And the collection method with the highest reliability was selected for subsequent detection by comparing with the flowering rate measured manually. The artificial measurement of the flowering rate was calculated by the five-point sampling method on the flowering of each wheat spike in the plot. The field experimental process is displayed in **Figure 15**. The area of each field plot was 1.2 × 1.2 m, and it took about 12s for the acquisition platform to obtain data from a single plot. During the field experiment, the camera was mounted on the side of the acquisition platform and tilted 45 degrees to obtain wheat data. When the Angle of overhead shooting is too large, the morphological structure of the distant spikelets and florets will be too small to be recognized, resulting in decreased accuracy. Due to this, the data obtained was partial plot images in the field. The three data acquisition methods of 0.5s interval shooting, 1s interval shooting, and video acquisition were used to determine the flowering period for field wheat. Due to the different time intervals of image data acquisition, the count of florets and spikelets identified by the improved YOLOv5s model was different under different acquisition methods. About 25 images were acquired in a single plot using the 0.5s interval shooting method, and about 13 images were acquired in a single plot using the 1s interval shooting method. The spikelets and florets detected under the three acquisition methods were superimposed respectively, and the determination of the wheat flowering period was performed based on the method mentioned in subsection 2.3. There was overlap between two adjacent image frames obtained. Repeated counting was used to reduce the influence of objective factors such as wind, wheat leaf occlusion, and the vibration of the acquisition platform during field data collection, which would lead to the failure of detection of some targets and decrease the accuracy. The comparison results of field experiments under different acquisition methods are shown in **Table 7**. Therefore, the least number of florets and spikelets were detected by the improved YOLOv5s model using the 1s interval shooting method compared with the others.

**Table 7:** The flowering period determination under different collection methods.

Collection method	Manually calculate flowering rate/%	Algorithm statistical results				Calculation error (%)
		Floret count	Spikelet count	Flowering rate (%)	Flowering period determination	
Video acquisition	9.3	2214	17407	12.7	heading period	3.4
0.5s interval shooting		87	1261	6.9	heading period	**2.4**
1s interval shooting		28	592	4.7	heading period	4.6
Video acquisition	32.4	7198	17951	40.1	heading period	7.7
0.5s interval shooting		351	1240	28.3	heading period	**4.1**
1s interval shooting		175	676	25.9	heading period	6.5
Video acquisition	57.7	15498	23482	66.0	flowering period	8.3
0.5s interval shooting		891	1635	54.5	flowering period	**3.2**
1s interval shooting		448	843	53.1	flowering period	4.6
Video acquisition	87.2	19813	20489	96.7	flowering period	9.5
0.5s interval shooting		1265	1474	85.8	flowering period	**1.4**
1s interval shooting		665	796	83.5	flowering period	3.7

(The manually calculated flowering rate as the actual flowering rate).

Under different flowering conditions, the flowering rates calculated manually of wheat and calculated by the improved YOLOv5s model under different acquisition methods were compared and analyzed. When the 0.5s interval shooting method was used for data acquisition, the error between the calculated flowering rate of the improved YOLOv5s model and the actual flowering rate was 2.4%, 4.1%, 3.2%, and 1.4% as shown in **Table 7**, respectively. It can be inferred that the 0.5s interval shooting method has higher reliability than the other two methods and it is used for subsequent detection. When the actual flowering rate exceeds about 30%, the error was negatively correlated with the actual flowering rate. When the actual flowering rate was about 0 to 30%, the error was positively correlated with the actual flowering rate. Inspection by analysis result revealed that the order of wheat flowering was from middle to top, and finally bottom. The middle spikelets develop faster, bloom, and pollinate first, and the anther size is similar to the bottom anther but slightly larger than the top anther. At flowering rates approaching 30%, there will be more apical florets of small size and not obvious, which is easy to cause florets to be missed identification, so the error increases. The characteristics of florets become more obvious with the increase in flowering rate, and the missed detection rate also decreases. Therefore, the error is negatively correlated with the flowering rate when the flowering rate exceeds about 30%.

The flowering rate calculated by video detection is higher than that calculated manually in the three collection methods. The dynamic characteristics of floret changed little while that of spikelets changed strongly with the operation of the phenotypic platform in the field by analyzing the detection video frame-by-frame. In images with several frames apart, the continuous capture of florets was better, and spikelets were missed, so the calculated flowering rate was higher than that calculated manually. Compared with the method with the smallest error of 0.5s interval shooting, the error change of the detection results of the method with the 1s interval shooting is similar to that of the former. However, the prediction results were more likely to be affected by accidental factors due to the lower collection frequency, so the error is slightly greater than the detection method collected once every 0.5s interval.

## 4 Conclusion

The flowering period of wheat is one of the key agronomically valuable traits. To realize the real-time determination of the flowering period of wheat images in the field, a determination method based on the improved YOLOv5s model was proposed. Finally, the accurate detection of florets and spikelets was achieved, and the real-time determination of the wheat flowering period was completed based on the ratio of florets and spikelets. By fusing FF, CBAM, and TSDH, the improved YOLOv5s model attenuated the distortion of the extracted feature images caused by light, and solve the problem of obscured florets, spikelets missed detection, and difficult to be detected small targets in population images.

The proposed improved YOLOv5s model improved the accuracy of floret and spikelet recognition, with accuracy of 88.9% and 96.8%, respectively. The average detection time of a single image was 11.5ms. The average detection accuracy was higher than 86.4% under the complex conditions of strong light, drastic angular distortion, blurred target area, serious occlusion, and abundant population phenotype. The effectiveness and adaptability of the model in a variety of complex situations were proved. By conducting an ablation experiment to investigate the contribution of various strategies to model improvement. Among them, the Feature fusion method, the CBAM, and the integrated transformer multi-detection header structure showed an improvement in the mean average accuracy of the model by 8.3%, 8.1%, and 6.0%, respectively. Finally, the field experiment was carried out and the overall flowering rate of the plot was estimated based on the proportion of florets to spikelets. Compared with the results of artificial measurement of the flowering rate, the error of the model was less than 5% compared with the actual detection of the flowering rate, and the determination accuracy of the flowering period reached 100%, which meets the demand of practical application and demonstrates the feasibility of the research.

Our future work will focus on how to improve the detection speed of the model on the basis of ensuring the detection performance. At the same time, an efficient and accurate method for the whole growth period of wheat will be studied and applied to the breeding process, so as to provide data support for the improvement of the stable yield of wheat.

## Data availability statement

The raw data supporting the conclusions of this article will be made available by the authors, without undue reservation.

## Author contributions

PL, XS, and LL planned and designed the research, and wrote the manuscript. PL and XL advised on the design of the model and revised the manuscript. PL, XL, and CW supervised and revised the manuscript. WZ, YL, and JZ performed experiments partially. All authors contributed to the article and approved the submitted version.
